# TD-Det: A Tiny Size Dense Aphid Detection Network under In-Field Environment

**DOI:** 10.3390/insects13060501

**Published:** 2022-05-26

**Authors:** Yue Teng, Rujing Wang, Jianming Du, Ziliang Huang, Qiong Zhou, Lin Jiao

**Affiliations:** 1 Institute of Intelligent Machines, Hefei Institutes of Physical Science, Chinese Academy of Sciences, Hefei 230031, China; yueteng@mail.ustc.edu.cn (Y.T.); djming@iim.ac.cn (J.D.); zlh94@mail.ustc.edu.cn (Z.H.); zhoujoan@mail.ustc.edu.cn (Q.Z.); 2 Science Island Branch of Graduate School, University of Science and Technology of China, Hefei 230026, China; 3 College of Information and Computer, Anhui Agricultural University, Hefei 230036, China; 4 School of Internet, Anhui Univiersity, Hefei 230031, China

**Keywords:** aphid detection, tiny size, dense distribution, multi-viewpoint detection, convolution neural network, transformer, multi-resolution training

## Abstract

**Simple Summary:**

Accurate recognition and detection of pests is the basis of integrated pest management (IPM). Manual pest detection is a time-consuming and laborious work. We use computer vision technology to design an automatic aphid detection network. Compared with other methods, our model can improve the performance and efficiency of aphid detection simultaneously. Experimental results prove the effectiveness of our method.

**Abstract:**

It is well recognized that aphid infestation severely reduces crop yield and further leads to significant economic loss. Therefore, accurately and efficiently detecting aphids is of vital importance in pest management. However, most existing detection methods suffer from unsatisfactory performance without fully considering the aphid characteristics, including tiny size, dense distribution, and multi-viewpoint data quality. In addition, existing clustered tiny-sized pest detection methods improve performance at the cost of time and do not meet the real-time requirements. To address the aforementioned issues, we propose a robust aphid detection method with two customized core designs: a Transformer feature pyramid network (T-FPN) and a multi-resolution training method (MTM). To be specific, the T-FPN is employed to improve the feature extraction capability by a feature-wise Transformer module (FTM) and a channel-wise feature recalibration module (CFRM), while the MTM aims at purifying the performance and lifting the efficiency simultaneously with a coarse-to-fine training pattern. To fully demonstrate the validity of our methods, abundant experiments are conducted on a densely clustered tiny pest dataset. Our method can achieve an average recall of 46.1% and an average precision of 74.2%, which outperforms other state-of-the-art methods, including ATSS, Cascade R-CNN, FCOS, FoveaBox, and CRA-Net. The efficiency comparison shows that our method can achieve the fastest training speed and obtain 0.045 s per image testing time, meeting the real-time detection. In general, our TD-Det can accurately and efficiently detect in-field aphids and lays a solid foundation for automated aphid detection and ranking.

## 1. Introduction

Aphid infestation seriously reduces grain yield by soaking up plant juices and transmitting wheat virus disease. Pesticides are often used to deal with pest infestations [1,2]. However, the overuse and misuse of pesticides lead to environmental degradation and food safety issues. Accurately and efficiently detecting pests is the foundation of integrated pest management (IPM) [3]. Due to the manual recognition and location being a time-consuming and laborious work, researchers attempt to solve this problem by computer vision techniques. Traditional machine-learning-based algorithms [4,5,6,7] identify specific pests by hand-designed feature extraction methods, which result in inadequate generalization for practical application. Since the ImageNet Large-Scale Visual Recognition Challenge (ILSVRC) [8], deep-learning-based methods obtained state-of-the-art (SOTA) performance in general object detection. Therefore, researchers transfer the deep-learning-based detector to pest recognition and location.

Rustia et al. used insect sticky paper traps and wireless imaging devices to construct a greenhouse dataset for detecting and recognizing pests in a fixed environment [9]. With light-trap devices, Liu et al. designed a pest detector by using global and local activation features to recognize and localization 16 species from 2 orders, including Lepidoptera and Coleoptera [10]. Jiao et al. proposed an anchor-free network to identify and locate pests of 24 types, but the incomplete feature fusion resisted the improvement in the detection performance [11]. Subsequently, a sampling-balanced region proposal network was designed to improve the performance of small-size pests by introducing an attention mechanism into the residual network (ResNet) [12] for obtaining richer pest feature appearances [13]. Aimed at the small-size high-similarity pest detection problem, Dong et al. designed a CRA-Net to improve the feature extraction capability of the CNN-based method with a channel recalibration feature pyramid network and an adaptive anchor module [14].

The light-trap methods automatically detect crop pests by using light-trap devices, but the expensive equipment overhead limits the development of IPM. In addition, the above-mentioned methods accurately detect pests in a fixed background but are not suitable for the in-field environment because of the complex lighting, various shooting angles, different image quality, and intricate background. Due to the limitation of light-trap methods, researchers tend to recognize and locate pests in the field environment. Wu et al. constructed a large-scale insect dataset IP102 including 75,000 images with 102 pest species, which laid the foundation of pest recognition and location [15]. Pattnaik et al. explored the feasibility of deep learning-based pest identification methods with the 10-class tomato pest dataset [16]. Ayan et al. combined different convolutional neural networks (CNNs) into a unified pest identification network and automatically selected the combination weight to carry out pest identification through the genetic algorithm [17]. Thenmozhi et al. explored the results of four deep-learning-based methods (AlexNet [8], ResNet [12], LeNet [18], and VGG [19]) on three pest datasets using the method of transfer learning [20]. Xie et al. used multi-task sparse representation and multi-kernel learning to identify 24-class common field pests [21].

The above methods use the CNN-based model to recognize pests in the simple in-field environment, in which most images consist of one or two pest close-ups. Although these methods obtain satisfactory performance, they lack practical application value. In the complex in-field environment, Wang et al. solved the difficulty of small-size pest recognition by combining the context-aware information (longitude, latitude, temperature, and humidity) with the Faster R-CNN [22]. Due to the clustering habits of pests, the real in-field data exhibit dense distribution. Li et al. proposed a coarse-to-fine network to recognize and detect aphids by combining the two-stage network and one-stage network into a uniform pipeline. The network used two-stage architecture to capture the region of aphids and employed another fine network to detect aphids by regarding the region from the two-stage network as a new image, which results in inadequate timeliness [23]. Subsequently, a data augmentation method was designed to improve the detection performance of multi-scale and multi-attitude pests. It expanded data by rotating and scaling in the training phase and detected pests with multi-resolution images in the testing phase. This method improved the performance but ignored the time cost regardless of the training and testing phase resulting in inadequate practical application ability [24]. Du et al. defined the problem of densely clustered tiny pest detection and proposed an aphid detector that used a cluster region proposal network to find the region of aphid and employed a local detector group to recognize each aphid by transforming the aphid region to a single image [25]. The method could accurately detect aphids but the significantly slow test speed limited the practical application. Due to the detection difficulty of tiny-sized dense pests in the real field environment, existing methods improve performance at the cost of time. In addition, the incomplete feature enhancement capacity of existing methods results in inadequate performance improvement in aphids detection.

**Figure 1:**
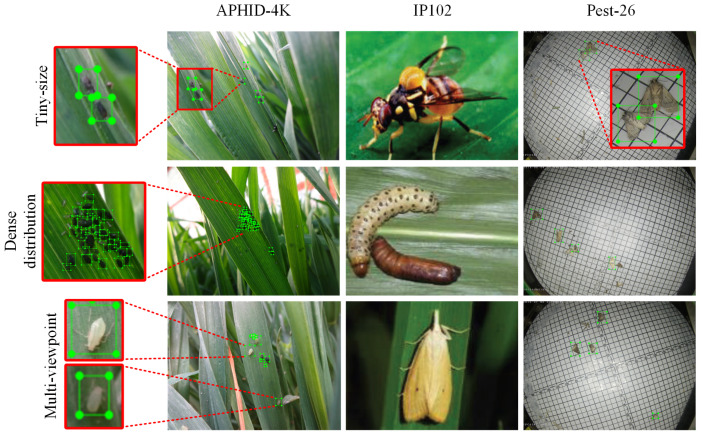
The comparison of the APHID-4K and other pest datasets.

In summary, aphid data have three characteristics, including tiny size, dense distribution, and multiple viewpoints. Figure 1 shows the characteristics of aphid detection compared with other pest datasets (simple in-field environment dataset IP102 [15] and light-trap pest dataset Pest-26 [26]). Firstly, the average relative size of the APHID-4K dataset is 0.067%, which is significantly less than IP102 (37.622%) and Pest-26 (2.674%). Secondly, pests living in groups result in the situation of dense distribution on the APHID-4K dataset. The APHID-4K has an average of 12.60 aphid objects per image, which is significantly more than IP102 (1.17 pests per image) and Pest-26 (6.73 pests per image). Thirdly, due to the focusing difficulty of the data-collection device, multi-viewpoint aphids (aphids with varying degrees of clarity) exist in images.

Due to the above-mentioned characteristics, existing methods have a couple of limitations in aphid detection: (1) Due to the tiny size characteristics of aphids, the feature can difficult to extract, resulting in unsatisfactory detection performance [11,14,26]. The tiny-sized features gradually disappear in the process of convolution operation and the misty features are not satisfied with the accurate location of dense distribution aphids. (2) Due to multi-viewpoint aphids in the image, vague aphids will be missed. (3) Due to the dense distribution, existing methods have to detect the same aphid image multiple times, even finely detecting the aphid region as a new image resulting in inadequate practical application value (improve performance without considering efficiency) [23,24,25]. To solve the above-mentioned defects, we design a tiny-size dense aphid detection network (TD-Det) to improve the performance and efficiency simultaneously with two core designs: a Transformer feature pyramid network (T-FPN) and a multi-resolution training method (MTM). The T-FPN improves the feature expression ability of tiny-sized dense aphids by a feature-wise Transformer module (FTM) and a channel-wise feature recalibration module (CFRM), while the MTM is designed to train networks more robust (accuracy and efficiency) by using a coarse-to-fine resolution setting without extra time cost. In addition, extensive experiments on the APHID-4K dataset verify the feasibility of this study, and the results show that this study can improve detection performance and training efficiency. Ablation experiments show that our T-FPN and MTM can improve the detection performance of other methods in a plug-and-play manner.

## 2. Materials and Methods

### 2.1. Dataset

The in-field pest datasets usually adopt mobile phones or handheld data acquisition devices to collect pest images [27]. For tiny-sized dense distribution pest detection, some research constructed corresponding datasets [23,24,25]. To research the problems of tiny-sized dense distribution detection more equitably and effectively, we use APHID-4K as the experimental dataset. The APHID-4K includes 4294 images, and the resolution range is from 1440 × 1080 to 4640 × 3480. The aphids are annotated using the top-left and bottom-right coordinates and the format of annotation files is XML, such as the PASCAL-VOC [28]. The composition of APHID-4K is shown in Table 1.

### 2.2. Methodologies

The in-field pest detection task involves two requirements: accuracy and real-time. (1) We hope that the detector can recognize all the pests in an image, rather than precise positioning. Even the non-precise bounding box can be accepted because, in IPM [3], the number of pests in an image is more important than the precise location. (2) Due to portable devices (mobile phones or portable data-collection devices [27]) being usually used to investigate crop growth, efficiency is also a core requirement. However, existing methods have difficulty satisfying the performance and efficiency simultaneously because of the characteristic of in-field pest data. The next best thing is existing methods [23,24,25] that improve detection performance at the cost of time, resulting in insufficient practical application ability.

**Figure 2:**
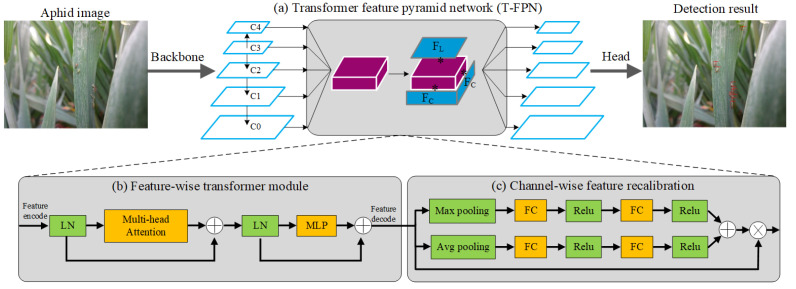
The network architecture of TD-Det with T-FPN, where LN is layer normalization, MLP is multi-layer perceptron, FC is fully connected, ReLu is rectified linear activation function, and C0–C4 are feature maps.

Therefore, we design a tiny-sized dense distribution aphid detection network (TD-Det) to detect aphids accurately and efficiently in the field environment. The TD-Det includes two core designs: a Transformer feature pyramid network (T-FPN) and a multi-resolution training method (MTM). The T-FPN is employed to improve the feature extraction capability of networks, and the MTM is applied to improve the performance with faster training time. Specifically, the network architecture of TD-Det includes a backbone feature extraction network [12], a Transformer feature pyramid network (T-FPN), and a detection head network [29], as shown in Figure 2. Firstly, the backbone network is used to obtain feature maps from aphid images. Secondly, the T-FPN is employed to enhance tiny-sized, dense distribution aphid features by a feature-wise Transformer module and a channel-wise feature recalibration module. Thirdly, the detection head network is utilized to obtain classification and location results.

#### 2.2.1. Transformer Feature Pyramid Network (T-FPN)

When manually recognizing a blurry pest (hard sample) in an image, we consider surrounding pests to be homogeneous pests because of the clustered living habits of in-field pests. Due to the limitation of the receptive field, recent CNN-based pest detection methods only consider the features but ignore the clustering and interactions of pests. Unlike the CNN-based model, the Transformer model focuses on global information in the field of natural language processing [30]. Inspired by this, we design a Transformer feature pyramid network (T-FPN) to improve aphid detection performance with a feature-view Transformer module and a channel-wise recalibration module.

The FPN [31] uses top-down adjacent feature fusion to promote feature extraction for general object detection. However, in aphid detection, the tiny-sized features gradually disappear in the process of feature extraction (backbone), resulting in blurry semantic information misled by the bottom texture information via FPN. Therefore, we fuse all the features into a unified feature map and use the fused feature as the input of the feature-wise Transformer and the channel-wise recalibration to ensure efficiency.

Specifically, we use bilinear interpolation to resize the C2–C4 feature maps to the size of the C1 feature map and use 3 × 3 convolutions to resize the C0 feature map to the size of the C1 feature map. After the resize operation, we use the concat operation to fuse features. For feature map $C_{i}$, the size is $\left(w_{i},h_{i},d_{i}\right)$, where the $w_{i},h_{i},d_{i}$ is the width, high, and depth (channel), respectively. We stack the resized features C0–C4 at the dimension of the channel (depth), and the size of fused feature is $\left(w_{1},h_{1},5\times d_{1}\right)$. Then, 1 × 1 convolutions are used to change the channel number to the original size $d_{1}$. We choose the C1 feature map rather than the C2 feature map to balance the performance and efficiency because the size of pests is small. The feature fusion method improves the feature expression ability and reduces the gap between semantic information and texture information. Meanwhile, using the feature-wise Transformer module and the channel-wise recalibration module on the fused feature only once ensures sufficient efficiency.

#### 2.2.2. Feature-Wise Transformer Module (FTM)

Transformer technology has been successfully used in machine vision [32,33]. However, these methods need lots of memory, while using Transformer technology in backbone networks results in insufficient efficiency because the bigger image size brings a lot of computation. Although the Swin Transformer [33] has improved efficiency by calculating attention information in each patch and conveying attention information using a few key points, information loss is essential in the transmission process, resulting in degraded performance. Therefore, we design a feature-wise Transformer module to calculate attention information on the whole feature map to provide sufficient efficiency and accuracy.

With the fused feature, we design a feature-wise Transformer module to improve the aphid detection performance, as shown in Figure 2b. the feature-wise Transformer module includes a feature encode/decode, layer normalization (LN), multi-head attention, and a multi-layer perceptron (MLP). For the fused feature $F_{x,y,c}$, where (x,y) is the horizontal position coordinate of the feature map, and *c* is the channel number. We shift the size to $V_{x\times y,c}$ using feature encoding. After LN [34], we put the $V_{x\times y,c}$ into the multi-head attention, as shown in Formula (1):

(1) $$Attention\left(Q,K,V\right)=softmax\left(\frac{Q^{T}\;K}{\sqrt{d_{k}}}\right)\cdot V$$

where *Q*, *K*, and *V* are the results of $V_{x\times y,c}$ through linear mapping, ${\left[\cdot \right]}^{T}$ is transpose operations, and $d_{k}$ is the dimensionality of *K* (here is the channel number). The multi-head attention uses the linear layer to map (Q,K,V) to different distance spaces, and the attention mechanism enhances the fuzzy aphid feature with other aphid features in the image. Subsequently, the MLP maps the attention information to the original distance space.

#### 2.2.3. Channel-Wise Feature Recalibration Module (CFRM)

SENet [35] is a convolutional neural network, which uses a channel-attention mechanism to calculate channel weights for improving feature extraction capability. However, the incomplete attention is not satisfied with tiny-sized dense distribution aphids. For a fused feature map F(x,y,c), where x,y is the point of feature and the *c* is the feature channel. After the feature-wise Transformer, the point-wise feature has been improved. Therefore, we use a channel-wise recalibration to improve channel-wise feature expression ability and combine the feature-wise Transformer to comprehensively improve performance.

After the feature-wise Transformer module, we use max pooling and average pooling to obtain the channel value and the full connection (FC) is utilized to calculate the relation between each channel. The learned weights are multiplied by feature maps, as shown in Figure 2c. After feature-wise Transformer and channel-wise recalibration, we use bilinear interpolation and 3 × 3 convolutions to resize the fused feature to the size of the original feature map. In addition, our T-FPN can improve the detection performance in a plug-and-play manner and can combine simply with other detectors.

#### 2.2.4. Two Versions of TD-Det

To increase the application value, we design two versions of TD-Det, including the real-time version (RV) and the precision version (PV). The TD-Det with PV pays more attention to precision and the TD-Det with RV balances the accuracy and efficiency. The distinction between PV and RV is the different selection of feature maps, in which the PV is partial to using the bottom layer features and the RV is inclined to use top layer features. Specifically, the RV version uses the C1–C5 features as the input to the T-FPN, and the PV version uses the C0–C4 features as the input to the T-FPN. The experimental results in Section 3.4 show that the RV already has more accuracy and efficiency than other methods, and the PV is more accurate than RV.

#### 2.2.5. Multi-Resolution Training Method (MTM)

Different from other tiny-sized datasets such as TinyPerson [36], aphid images are mostly taken at the micro-focal length, resulting in multi-viewpoint objects in the same image. This causes degraded performance in two situations: (1) one detected bounding box contains multiple aphids and (2) a large number of undetected fuzzy aphids. Therefore, we design a multi-resolution training method (MTM) to improve the detection performance with higher efficiency.

**Figure 3:**
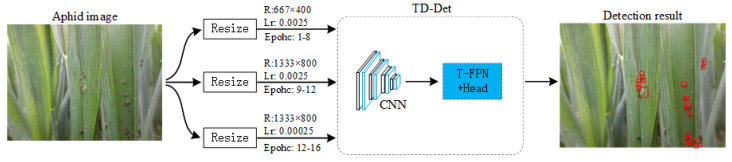
The architecture of the multi-resolution training method (MTM), where R is the resolution of images, Lr is the learning rate, and the red bounding boxes are detected aphids.

The MTM uses a coarse-to-fine resolution setting to train the network in the form of augmenting low-resolution aphid data by resizing high-resolution images, as shown in Figure 3. In general object detection, we resize the variably sized images to a given size (COCO [37] is 1333 × 800, and PASCAL VOC [28] is 1000 × 600) for uniform network training. The machine-made resizing operation cannot change the resolution discrepancy of aphid objects. Our MTM improves the performance of low-resolution aphid predictions by using the coarse-to-fine resolution setting. Specifically, we first resize all images to a low resolution (667 × 400) for training 8 epochs. The training time is much less than the original resolution due to the reduced image size. Then, we resize all images from the original to high resolution (1333 × 800) for training high-resolution images using 4 epochs. Finally, we reduce the learning rate by 0.1 times to fine-tune 4 epochs, similar to common methods. Our MTM is a practical method that could improve detection performance with higher efficiency.

### 2.3. Loss Function of TD-Det

For training our TD-Det, we design the loss function including classification loss, center-ness loss, and regression loss, as shown in Formula (2). In the test phase, we multiply the center-ness branch to the regression branch to ensure points situate the center of the prediction bounding box:

(2) $$L_{total}=L_{cls}+L_{reg}+L_{center}$$

(3) $$L_{cls}=\frac{1}{N_{pos}}\sum_{i=1}^{N_{cls}}FL\left(p_{i},\hat{p_{i}}\right)$$

(4) $$L_{reg}=-In\frac{Intersection(B,\hat{B})}{Union(B,\hat{B})}$$

(5) $$L_{center}=BCE\left(centerness,\hat{centerness}\right)$$

where $L_{cls}$ is the focal loss [38], $L_{reg}$ is the Intersection over Union (IoU) loss [39], $L_{center}$ uses binary cross entropy loss, and the target of center-ness is followed by the fully convolutional one-stage object detection (FCOS) [29].

## 3. Experiments and Discussions

### 3.1. Experiment Settings

The backpropagation and Stochastic Gradient Descent (SGD) [40] are employed to train our TD-Det. In the training phase, each SGD mini-batch is constructed from a single pest image that contains 256 samples with the ratio of 1:1 selected between negative samples and positive samples. Gaussian distribution with a mean of 0 and a standard deviation of 0.01 is used to initialize the parameters of the classification regression layer. We train a total of 16 epochs with a Momentum of 0.9, among which the first 12 epochs have a learning rate of 0.0025, and the last 4 epochs are 0.00025. With the MTM, the resolution of 667×400 is set in the first 8 epochs, and the resolution of 1333×800 is set in the last 8 epochs. Our experiment is deployed on a Dell 750 server with NVIDIA Titan RTX GPU (24G memory) using the Mmdetection2.0.0 [41] framework and Python 3.8. Unless otherwise stated, all of the methods use ResNet50 as the backbone network and use the same parameter settings.

### 3.2. Evaluation Metrics

IoU is the foundation of detection evaluation and is defined as Formula (6):

(6) $$IoU_{a,b}=\frac{area(a)\cap area(b)}{area(a)\cup area(b)}$$

where area(·) is the area of the region in an image. We use true positive (TP), false positive (FP), true negative (TN), and false negative (FN) to determine the results of the prediction. Precision and recall are defined as Formulas (7) and (8), respectively:

(7) $$Precision=\frac{TP}{TP+FP}$$

(8) $$Recall=\frac{TP}{TP+FN}$$

To evaluate models comprehensively (accuracy ratio and recall ratio), we use average precision (AP), as shown in Formula (9):

(9) AP(c)=∫Precision(c)dRecall(c)

where *c* is the category. The function graph of precision with respect to recall is the precision–recall (PR) curve. mAP is the mean AP of all categories, and $AP_{50}$ is the AP with IoU=0.5. Due to the ground truth being annotated by manual means, the precision of annotated bounding boxes has the situation of deviation. In addition, the number of aphids is more important than the precise positioning. Therefore, the $AP_{50}$ index is more reference value than $AP_{75}$ and mAP (general object detection dataset PASCAL VOC [28] use $AP_{50}$ index only).

In addition, we use *P_training* and *P_testing* to show the practicability of detectors, as shown in Formulas (10) and (11), respectively. The values of *P_training* and *P_testing* display the practical application value of networks, and the higher values illustrate the more accurate performance and higher efficiency:

(10) $$P\_training=AP_{50}/training\_time$$

(11) $$P\_testing=AP_{50}/testing\_time$$

### 3.3. Contrastive Methods Involved in Experiments

We compared the performance of our method with Faster R-CNN [31,42], Libra R-CNN [43], ATSS [44], Cascade R-CNN [45], FCOS [29], Retinanet [38], FoveaBox [46], CRA-Net [14], and DCTDet [25]. Among them, Faster R-CNN is the baseline of two-stage object detection, and Libra R-CNN, Cascade R-CNN, and ATSS are the improved two-stage object detection. The FoveaBox, FCOS, and RetinaNet methods are one-stage object detection methods, and CRA-Net and DCTDet are existing pest detection methods.

### 3.4. Performance on the APHID-4K Dataset

The performance of the networks are shown in Table 2. Following experimental results, two-stage networks almost outperform one-stage networks. However, our TD-Det (a one-stage network) outperforms all methods, even the real-time version. The precision version of TD-Det obtain 74.2% $AP_{50}$ and 46.1% mRecall on the APHID-4K dataset, 15.9% and 27.4% higher than FoveaBox, and 9.0% and 46.4% higher than CRA-Net detector.

Furthermore, Table 3 shows the efficiency comparison with other methods. Our TD-Det (RV) achieves the fastest training speed of 0.045 s/iter and the highest P_training of 9.55%/s. The TD-Det (PV) achieves the best performance, and the test speed of 0.1 s/img meets real-time requirements. For the TD-Det (RV), the value in practical application is much higher than FCOS, although the test speed of 0.045 s/img is slightly lower than the FCOS of 0.041 s/img. In general, our TD-Det, either the real-time version or the precision version, outperforms other methods and achieves state-of-the-art (SOTA) results.

Due to the MTM improving the performance of detectors without extra testing time, the *P_testing* is increased by $AP_{50}$ value. In the training phase, the MTM reduces the training time, and the *P_training* is increased by $AP_{50}$ value and the training time simultaneously. Due to the unusable acceleration of MTM in the testing phase, the *P_testing* of our TD-Det is inferior to FCOS. However, the 8.16% improvement in performance is more important than the 1.49% *P_testing* decline. By comprehensive comparison, our TD-Det achieves the best performance and efficiency.

### 3.5. Ablation Experiments

**Performance of T-FPN with various networks.** We compare the performance of T-FPN with other methods in a plug-and-play manner, as shown in Table 4. The detection performance of all methods is improved by using our T-FPN, which shows its practical application value. The T-FPN can improve the $AP_{50}$ from 0.4% to 1.1% with Cascade R-CNN and FoveaBox, respectively. The experimental results show that our T-FPN can help networks to improve feature extraction capability and performance.

**Performance of MTM with various detection methods.** We compare the performance of MTM with various networks as shown in Table 5. Due to the simple structure of one-stage networks having difficult extracting fine features, the improvement of one-stage networks is higher than two-stage networks. This illustrates that the coarse-to-fine training pattern can help networks to obtain fine-grained features. The improved values of mRecall show that our MTM can improve detection results of fuzzy aphids by resizing high-resolution images to low-resolution images. Because the low-resolution images are trained faster than the high-resolution images, the training time of all networks is reduced by using our MTM. The experimental results show that our MTM can improve the performance and shorten training time simultaneously for both two-stage networks or one-stage networks.

**Backbone of our TD-Net.** Due to the requirement of aphid detection paying more attention to position rather than high-value IoU, the $AP_{50}$ is more important than $AP_{75}$ and AP. The performance of ResNet50 [12] is better than ResNet101 and ResNexts because the tiny-sized aphid feature gradually disappears in the process of the convolution operation, as shown in Table 6. For fairness, we choose the ResNet50 as the backbone of all the methods.

### 3.6. Analysis and Discussion

**Figure 4:**
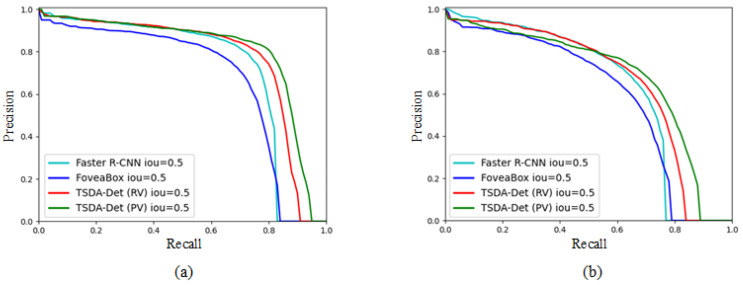
PR curve with IoU = 0.5. (**a**) PR curve of Macrosiphum avenae. (**b**) PR curve of Rhopalosiphum padi.

**PR curve.** To analyze the performance of our TD-Det in detail, we show the PR curve in Figure 4. Due to the $AP_{50}$ being more important than other indices, we show the PR curve under $IoU_{50}$. Following the PR curve, the two-stage network, Faster R-CNN, outperforms the one-stage network, FoveaBox. Our TD-Det models (either PV or RV) outperform other detectors. The advantage of our TD-Det models is that they reflect more accuracy in the region of high-value recall, which means that our TD-Det can provide more accurate results, ruling out missing detection.

**Figure 5:**
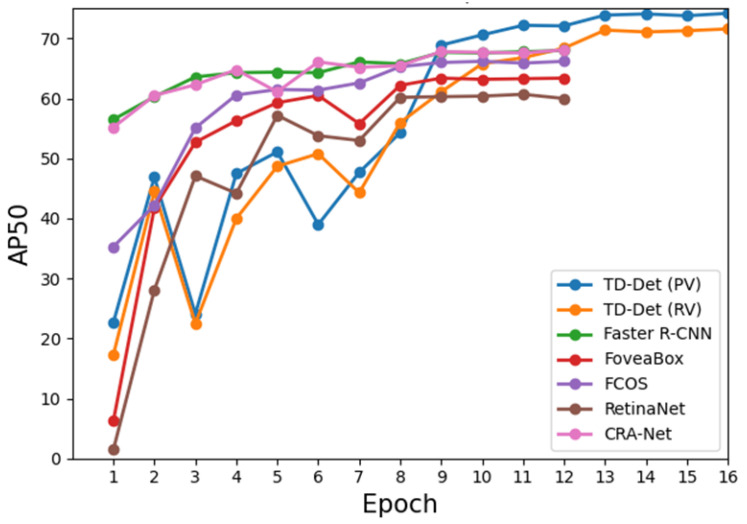
Performance comparison of $AP_{50}$.

**Performance comparison of each epoch.** We compare the performance of each epoch as shown in Figure 5. The $mAP_{50}$ curve of our methods shows the three-level performance improvement caused by the enhanced resolution and reduced learning rate. In addition, our method obtains start-of-the-art (SOTA) performance without reducing the learning rate at epoch 11 and epoch 12. Our methods (TD-Det (PV) with T-FPN and MTM) could effectively improve the detection performance of tiny-sized dense aphids.

### 3.7. Qualitative Results

**Figure 6:**
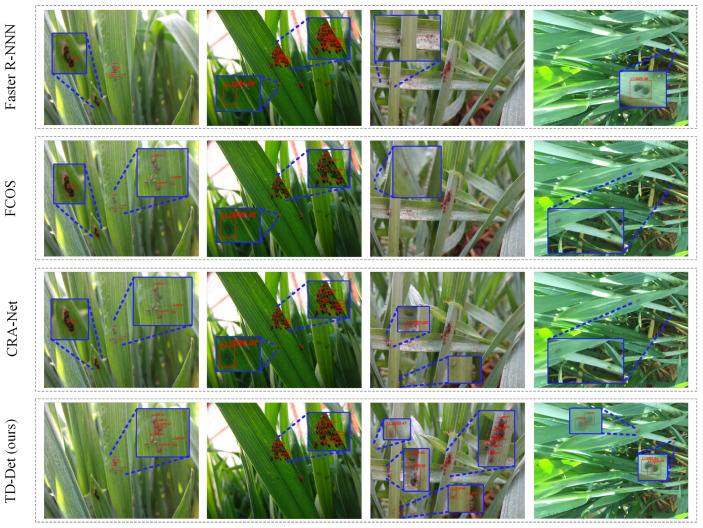
Comparison and visualization of detection results with other methods.

To visually observe the performance, we visualized the detection results of Faster R-CNN, FCOS, CRA-Net, and TD-Det (ours), as shown in Figure 6. We choose the images with various difficulty degrees, in which the first two columns exhibit the dense aphid images and the last two columns display the complex background images. In all scenarios, FCOS and CRA-Net can inadequately detect aphids, as shown in the second and third lines of Figure 6. Due to the manual super-parametric setting being inadequate for aphid detection, the Faster R-CNN has impertinent performance (one predicts bounding box with multiple aphids, and chaotic predicts the results), as shown in the first line of Figure 6. Our methods (TD-Det) acquire the best performance, as shown in the last line of Figure 6. In addition, in the first column of Figure 6, other methods misidentified other pests. For tiny-sized fuzzy aphid detection, the performances of other methods are inferior to that of TD-Det, as shown in the last columns of Figure 6.

## 4. Conclusions

Integrated pest management (IPM) requires specialized agricultural technicians, resulting in high labor costs. Meanwhile, the control of pests depends on pesticides, while the situation of excessive pesticides and the misuse of pesticides brings environmental pollution and food safety problems. Therefore, this study combines computer vision with IPM to provide an accurate and efficient pest detection tools to replace manual work. Specifically, this paper aims to solve the problem of tiny size, dense distribution and multi-viewpoint aphid detection. We propose a tiny-sized dense aphid detection network (TD-Det) that includes two core designs: a Transformer feature pyramid network (T-FPN) and a multi-resolution training method (MTM). The T-FPN focuses on improving the recognition accuracy of tiny-sized dense distribution aphids by a feature-wise Transformer module (FTM) and a channel-wise feature recalibration module (CFRM). Due to the tiny-sized aphids bringing difficulties in image capturing, we propose a multi-resolution training method (MTM) to improve the detection performance without extra time consumption. Furthermore, the MTM can improve training efficiency by using images with coarse-to-fine resolutions to train networks. Abundant experiments are performed on the APHID-4K dataset, and our method obtains 74.2% AP under the efficiency of 0.100 s per image. Ablation experiments demonstrate that our T-FPN and MTM can improve the performance of other detectors simply in a plug-and-play manner. In the future, we will focus on the research of real-time pest detection on mobile terminals to provide reasonable pest control suggestions to ordinary crop producers.

## Figures and Tables

**Table 1:** The constitution of the APHID-4K dataset.

		Training Images	Test Images	Training Aphids	Test Aphids
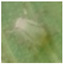	Macrosiphum avenae	2125	546	20,043	5203
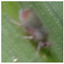	Rhopalosiphum padi	2093	507	23,074	5525

**Table 2:** Overall performance comparison.

Method	mAP	${\mathrm{AP}}_{50}$	${\mathrm{AP}}_{75}$	${\mathrm{AP}}_{s}$	${\mathrm{AP}}_{m}$	mRecall
*Other detectors*						
Faster R-CNN w/FPN [31]	26.1	68.0	13.1	21.9	30.1	36.7
Libra Faster R-CNN [43]	25.5	64.9	13.2	21.1	29.9	30.8
ATSS [44]	26.9	69.8	13.4	22.4	31.4	33.3
Cascade R-CNN [45]	27.3	69.3	14.1	23.4	31.0	38.3
FCOS [43]	24.9	66.2	11.3	19.9	29.3	32.3
RetinaNet [38]	21.7	60.0	9.4	15.4	26.7	37.1
FoveaBox [46]	23.1	63.4	10.1	18.2	27.7	36.2
CRA-Net [14]	26.1	68.1	13.0	21.8	30.1	31.5
DCTDet W/CCG [25]	27.1	68.5	13.7	22.0	30.4	32.8
*Ours*						
TD-Det(RV)	27.2	71.6	13.4	22.8	31.4	34.6
TD-Det(PV)	29.2	74.2	15.4	25.7	32.7	46.1

**Table 3:** The efficiency comparison.

Method	Training Time (s/iter)	Testing Time (s/img)	${\mathrm{AP}}_{50}$ (%)	mRecall (%)	*P_training* (%/s)	*P_testing* (%/s)	Parameters
*Other detectors*							
FPN Faster R-CNN [31]	0.111	0.048	68.0	36.7	6.13	14.17	41,353,306
Libra R-CNN [43]	0.118	0.050	64.9	37.4	5.50	12.98	41,616,474
ATSS [44]	0.106	0.048	69.8	40.3	6.59	14.54	32,115,532
Cascade R-CNN [45]	0.133	0.058	69.3	38.3	5.21	11.95	69,154,916
FCOS [43]	0.093	0.041	66.2	37.6	7.12	16.15	32,113,484
RetinaNet [38]	0.102	0.048	60.0	37.1	5.88	12.5	36,350,582
FoveaBox [46]	0.103	0.042	63.4	36.2	6.16	15.10	36,239,942
CRA-Net [14]	0.114	0.050	68.1	31.5	5.97	13.62	41,361,498
DCTDet [25]	0.280	0.213	68.5	32.8	2.45	3.22	84,706,732
*Ours*							
TD-Det(RV)	0.075	0.045	71.6	41.9	9.55	15.91	33,032,012
TD-Det(PV)	0.116	0.100	74.2	46.1	6.40	7.42	33,097,804

**Table 4:** The performance of various detection methods with or without T-FPN.

Method	T-FPN	AP	${\mathrm{AP}}_{50}$	mRecall
Faster R-CNN [31]		26.1	68.0	36.7
	√	26.6	68.4	37.2
Libra R-CNN [43]		25.5	64.9	37.4
	√	25.9	65.4	37.7
Cascade R-CNN [45]		27.3	69.3	38.3
	√	27.4	69.7	38.2
FCOS [29]		24.9	66.2	37.6
	√	25.0	67.1	37.4
RetinaNet [38]		21.7	60.0	37.1
	√	22.0	60.9	37.0
FoveaBox [46]		23.1	63.4	36.2
	√	23.5	64.5	36.3

**Table 5:** The performance of various detection methods with or without MTM.

Method	MTM	${\mathrm{AP}}_{50}$	mRecall	Training Time (s/iter)	Test Time (s/img)
Faster R-CNN [31]		68.0	36.7	0.111	0.048
	*√*	68.5	37.2	0.079	0.047
Libra R-CNN [43]		64.9	37.4	0.118	0.050
	*√*	66.2	38.3	0.084	0.050
Cascade R-CNN [45]		69.3	38.3	0.133	0.058
	*√*	69.4	38.5	0.102	0.058
FCOS [29]		66.2	37.6	0.093	0.041
	*√*	69.3	38.9	0.062	0.040
RetinaNet [38]		60.0	37.1	0.102	0.048
	*√*	62.8	38.2	0.072	0.048
FoveaBox [46]		63.4	36.2	0.103	0.042
	*√*	66.5	37.5	0.071	0.042

**Table 6:** The performance comparison of TD-Det with various backbones.

	Resnet50	Resnet101	Resnext50	Resnext101
** $AP_{50}$ **	74.2	74.0	73.2	72.9
** $AP_{75}$ **	15.4	14.5	14.4	13.9
** AP **	29.2	29.1	28.6	28.3

## Data Availability

The original contributions presented in the study are included in the article/supplementary materials, further inquiries can be directed to the corresponding author/s.
